# Deciphering the therapeutical potentials of rosmarinic acid

**DOI:** 10.1038/s41598-022-19735-y

**Published:** 2022-09-15

**Authors:** Sally El Kantar, Ali Yassin, Bilal Nehmeh, Louis Labaki, Sara Mitri, Fatima Naser Aldine, Aaron Hirko, Sergio Caballero, Eileen Monck, Alejandra Garcia-Maruniak, Elias Akoury

**Affiliations:** 1 Department of Natural Sciences, School of Arts and Sciences, Lebanese American University, Beirut, 1102-2801 Lebanon; 2 Inorganic and Organometallic Coordination Chemistry Laboratory, Faculty of Science, Lebanese University, Beirut, 1104 Lebanon; 3 Santa Fe College Perry Center Emerging Technologies, 14180 NW 119 Terrace, Alachua, FL 32615 USA

**Keywords:** Biochemistry, Biophysics, Chemical biology, Drug discovery, Diseases, Chemistry

## Abstract

Lemon balm is herbal tea used for soothing stomach cramps, indigestion, and nausea. Rosmarinic acid (RA) is one of its chemical constituents known for its therapeutic potentials against cancer, inflammatory and neuronal diseases such as the treatment of neurofibromatosis or prevention from Alzheimer’s diseases (AD). Despite efforts, recovery and purification of RA in high yields has not been entirely successful. Here, we report its aqueous extraction with optimal conditions and decipher the structure by nuclear magnetic resonance (NMR) spectroscopy. Using various physical–chemical and biological assays, we highlight its anti-aggregation inhibition potentials against the formation of Tau filaments, one of the hallmarks of AD. We then examine its anti-cancer potentials through reduction of the mitochondrial reductase activity in tumor cells and investigate its electrochemical properties by cyclic voltammetry. Our data demonstrates that RA is a prominent biologically active natural product with therapeutic potentials for drug discovery in AD, cancer therapy and inflammatory diseases.

## Introduction

*Melissa officinalis*, commonly known as lemon balm, is a therapeutic and culinary herb that belongs to the Labiatae family. Its major components include caffeic acid, rosmarinic acid, ferulic acid, and methyl carnosoate as well as other flavonoids^1,2^. Importantly, rosmarinic acid (RA) is a vital natural product reported for its antioxidant, anti-inflammatory, antiviral, and anti-allergic activities^3,4^. RA is used to treat peptic ulcers, cataract, rheumatoid arthritis, Herpes simplex^5,6^, arthrosclerosis^7^, and bronchial asthma^8–10^. Also, RA has been identified in therapeutic strategies for the prevention against neurodegenerative diseases, most importantly from Alzheimer’s disease (AD)^11,12^. AD is diagnosed by two hallmark aggregating proteins: intracellular Tau protein and extracellular neuritic plaques of amyloid Beta (Aβ) protein. Tau protein interacts physiologically with tubulin to stabilize and promote microtubule (MT) assemblies for transportation of vesicles and organelles. As AD advances, Tau protein becomes excessively phosphorylated, loses its ability to bind to MT and aggregates into Paired Helical Filaments (PHFs)^13^. This aggregation behavior is also stimulated in vitro by polyanions like RNA, heparin and acidic peptides^14^. Although there is still no contributing treatment or cure for AD, widespread research highlights the pathological consequences of these amyloidogenic formation and underlines therapeutic strategies through identification of natural products as inhibitors of Tau aggregation. Investigation of the mode of action of these disease-modifying drugs mainly involves identifying molecules that can either dissociate or prevent the formation of amyloids, their precursors and/or their derivatives. We have previously identified two families of molecules that inhibit Tau filament formation: Phthalocyanine tetrasulfonate (PcTS) and Phenothiazines. PcTS interacts with the tyrosine residues of Tau at specific aromatic interactions that are essential in trapping prefibrillar species^15^. On the other hand, phenothiazines specifically modify the cysteine residues of Tau and keep the protein in a monomeric disordered conformation thus preventing formation of filaments and their toxic precursors^16^. Equally important, the oligomeric and fibrillar aggregates of Aβ protein represent major molecular targets in drug discovery of AD. Accumulating research reported that RA binds directly to Aβ, inhibits its in vitro aggregation^17^ and delays disease progress in a Tg2576 AD mouse model^18^.

Remarkably, the use of natural products exhibiting anti-carcinogenic activities has been a wide field in cancer therapy^19^. A prominent natural product would modulate one or more of the various mechanisms of cellular proliferation, differentiation, apoptosis, angiogenesis, and metastasis of cancer cells. Carcinogenic components induce oxidative stress by production of excess reactive oxygen species (ROS), which results in apoptotic cell death after damaging DNA, proteins and cellular membranes^20^. RA scavenges radiation-induced ROS, prevents free radical-induced cell damage^21^ and provides a radioprotective effect against the ionizing radiation^22^. In a recent study, RA diminished Ultraviolet-induced damage in human keratinocytes which leads to skin cancer^23^. Similarly, RA reduced pancreatic cancer cells by suppression of cell viability/invasion and consequently the induction of cell apoptosis^24^. Accumulating evidence has implicated the responsibility of certain kinases in malignant transformation, inflammatory diseases, and several neuronal diseases such as neurofibromatosis, Alzheimer’s diseases, epilepsy, and depression^25^. Neurofibromatosis is a genetic disorder that causes tumor formation in peripheral nervous system resulting in growth and development defects of nervous tissues^26^. Plexiform neurofibroma is a variant that causes benign tumor and is present in 50% of children with type 1 neurofibromatosis^27,28^. Presently, there is no treatment for this disorder and the application of RA as an anti-cancer agent is plausible but requires more systematic research.

During the recent pandemic in 2020, therapeutic agents were identified against severe acute respiratory syndrome coronavirus 2 (SARS-CoV-2) infections, better known as Covid-19^29,30^ The angiotensin-converting enzyme 2 (ACE-2) has a major role in inflammation^31^ and is classified as a potent receptor for SARS-CoV-2^32^. A study reported that RA removes ROS from tissues and reduces inflammation by inhibiting the production of pro-inflammatory cytokines^33^. Therefore, RA is a potential candidate able to inhibit the expression of ACE-2^34,35^.

In spite of accumulating RA studies in the fields of neurodegeneration, anti-cancer and antibacterial properties; many results are not conclusive and contain no insights on the mode of action. On the other hand, the recovery and purification of RA from Lemon balm in high yields has not been entirely successful. Here, we report a highly efficient process for the aqueous isolation of RA from dried leaves of Lemon balm using Soxhlet extraction under optimum process conditions with high yield (96%) and elucidate its structure by NMR spectroscopy. We then investigated the effect of RA over heparin-induced Tau aggregation in vitro by using thioflavin T assay (ThT), atomic force microscopy (AFM), with various physical–chemical techniques and biological assays to highlight the properties of RA in anti-aggregation inhibition. While RA is also promising in drug discovery against different carcinoma, other types of cancer have not been explored. We also examined the inhibitory effects in plexiform neurofibroma tumor cells NF95. Moreover, the antibacterial activity of RA, like many phenolic compounds, could be attributed to several mechanisms of action such as membrane damage, inhibition of nucleic or cell membrane synthesis, and interaction with some essential enzymes^36^. However, the mechanism of action of RA is not fully understood despite evidence showing that the antibacterial activity of RA against *S. aureus* is due to the inhibition of surface proteins expression^37^. We therefore inspected the anti-bacterial activities by determining the minimal inhibitory concentrations using different bacterial strains.

## Materials and methods

### Isolation of RA

The optimal isolation method of RA has been done following several previous protocols^38,39^. Briefly, dried lemon balm leaves were finely grounded to a net weight of 140 g and introduced in a cellulose thimble for Soxhlet extraction. The sample was washed with 500 mL *n*-hexane for 10 h to discard all fatty materials and then refluxed twice with 1:1 H_2_O/ethanol mixture for 10 h. The resulting dark green solution contained RA and other natural compounds. The water-soluble compounds were discarded after *n*-butanol extraction and the dried material containing RA was then dissolved in 3:1 H_2_O/toluene solution to discard non-polar organic products. The pH of the water fraction was adjusted to 4.5 and fractionated twice with methyl tert-butyl ether (MTBE) to isolate RA from other organic compounds. MTBE was the most suitable solvent among others that were tested. The adjustment of pH was carefully controlled since RA is insoluble in water at pH 3.7. The dried material was then dissolved in 300 mL distilled water and partitioned twice with 100 mL toluene to isolate non-polar organic products from RA. The toluene upper level was discarded, and the pH of the water fraction was increased from pH 3.87 to 4.5 using 20 mM NaOH. At this pH, RA remains in water and other products are discarded after two extractions with 100 mL MTBE. The aqueous fraction was then adjusted to pH 3.75 and partitioned with five fractions of 100 mL MTBE. At this pH, RA becomes insoluble in water but soluble in MTBE. This latter fraction was collected and brought to dryness. The dried material was then dissolved in hot water, active charcoal added, and the solution stirred for 15 min filtrated and stored at 4 °C. The aqueous extraction of RA using Soxhlet distillation under optimal conditions was of high yield (96%).

### NMR spectroscopy

NMR experiments were acquired following standard protocols as previously reported^15,16^. NMR samples were prepared by dissolving 10 mg RA in 500 μL in deuterated methanol CD_3_OD. Spectra were internally referenced to the singlet resonance of 4,4-dimethyl-4-silapentane-1-sulfonic acid (DSS) at 0 ppm. Spectra were recorded on a BRUKER AVANCE DRX 400 MHz NMR spectrometer. All spectra were recorded using a sample with a concentration of 1.6 mM at 300 K. The experiments used were COrrelation SpectroscopY (COSY), Nuclear Overhauser Effect SpectroscopY (NOESY), Heteronuclear Single Coherence Correlation (^13^C-HSQC), Heteronuclear Multiple Bond Correlation (^13^C-HMBC). 1D ^1^H NMR parameters included 2 s acquisition time, 1.5 s relaxation delay, 16 scans and a total time of 1 min. Variations on the 1D experiment include 1D NOE, selective decoupling solvent saturation and T_1_ determination. 1D ^13^C NMR parameters included 0.8 s acquisition time with proton decoupling, 2.0 s relaxation delay with NOE enhancement, 192 scans and a total time of 10 min. All 2D NMR experiments parameters included 1.5 s relaxation delay, 1 to 8 scans and a total time between 5 min and 1 h.

### Aggregation assay

Heparin (average molecular weight of 3000) and thioflavin T (ThT) were obtained from Sigma (Sigma-Aldrich Chemie GmbH, Schelldorf, Germany). Full length Tau protein was expressed in *Escherichia coli* and purified using the protocol described previously^15^. Tau (50 μM) was incubated alone or in presence of 12.5 μM heparin in 20 mM BES buffer, pH 7.4, and 1 mM DTT for 10 min at 95 °C. The sample was replenished with 1 mM DTT, and a protease inhibitor mix (1 mM PMSF, 1 mM EDTA, 1 mM EGTA, 1 μg/mL leupeptin, 1 μg/mL aprotinin, and 1 μg/mL pepstatin) was added. RA (100 μM) was added to protein vials, and the solutions were incubated for 7 days at 37 °C. Assembly of PHFs was then quantitatively measured by fluorescence assay using thioflavin T (ThT). Before each fluorescence measurement, the samples were equilibrated for 30 min at room temperature and diluted sixfold with BES buffer followed by the addition of 20 μM ThT. Fluorescence was measured on a Safire (TECAN) fluorimeter with excitation and emission wavelengths of 412 and 490 nm, respectively. All samples were freshly prepared, and each measurement was repeated three times.

### Atomic force microscopy

Samples were diluted in PBS buffer and 100 μL of the dilution was deposited on a DETA surface. MFP-3D atomic force microscope (Asylum Research, Santa Barbara, Ca, USA) with cantilevers (Olympus BL-RC150VB, nominal spring constant 0.01 N/m, 20 nm tip radius) was used to image the samples in contact mode using a silicon nitride AFM cantilever tip (20 nm tip radius, nominal spring constant 0.01 N/m, Bruker AFM Probes, Camarillo, CA). Images were acquired at scanning rates ranging from 0.25 to 0.75 Hz with a 256 × 256-pixel image resolution.

### MTT assay for cell viability

NF95 and Schwann Cells were obtained from ATCC (Manassas, USA). Both cell lines were cultured in Dulbecco’s Modified Eagle Medium High Glucose (DMEM; Gibco, Grand Island, USA) with 10% Fetal Bovine Serum (FBS; SAFC, St Louis, USA). The cells were incubated at 37 °C and 5% CO_2_. Trypsin 0.25% (in HBSS, GenClone, San Diego, USA) was used for splitting cells. MTT Thiazolyl Blue Tetrazolium Bromide 98% (Sigma, St. Louis, USA) was used to assay the mitochondrial reductase activity of cells treated with RA. RA was suspended in Dimethyl sulfoxide (DMSO, Sigma, St. Louis, USA) at a concentration of 5 mg/mL. The MTT assay was performed to reflect cellular viability as previously reported^40,41^. Briefly, 100 μL of NF95 and Schwann Cells were seeded at 1 × 10^3^ cells/mL per well in a 96 cluster well plate and incubated for 24 h for cell attachment. After removing the media, the cells were treated with 200 μL of 0, 10, 20, 40, 80 and 160 μM of RA diluted in DMEM High Glucose media with five replicates per concentration, and incubated for 24, 48, and 72 h before treating them with MTT. For this, 20 μL of 5 mg/mL of MTT was added to each well and incubated for 4 h/37 °C. Finally, 100 μL of DMSO was added to each well, and after 10 min the colorimetric reduction from MTT (yellow) to formazan (violet) was analyzed using a spectrophotometer at OD 630 nm. Another set of blank wells with neither RA nor MTT treatments were added per plate ro monitor cell condition throughout the duration of the experiment.

### Antibacterial activity of RA

In this study, three Gram-positive bacterial isolates (*Staphylococcus aureus* MRSA, *Staphylococcus aureus* MSSA, *Streptococcus agalactiae*) and four Gram-negative bacterial isolates (*Klebsiella pneumoniae*, *Escherichia coli*, *Pseudomonas aeruginosa* and *Proteus mirabilis*) were tested. These clinical strains were provided by the Clinical Microbiology Laboratory at the Lebanese American University Medical Center–Rizk Hospital (LAUMC-RH, Beirut Lebanon). The well diffusion assay method was used to determine the antimicrobial activity of RA. Inoculums in 0.9% saline were prepared from freshly streaked Mueller–Hinton agar MHA (LabM Ltd, Bury, U.K.) plates to a 0.5 McFarland equivalent turbidity of bacteria. Inoculum of each bacterial culture was spread on 25 mm thick MHA plates. A 10.5 mm well was punched in the middle of each plate, using a cork borer, and filled with 200 μL of different concentrations of RA. The plates were then incubated in the upright position at 37 °C for 24 h. The next day, the zones of bacterial growth inhibition around the wells were measured in three different angles, if present. 15 concentrations of RA (0.05, 0.2, 0.4, 0.6, 0.8, 1, 5, 10, 12, 14, 16, 18, 20, 22 and 25 mg/mL) were tested against the 7 bacterial strains. For each concentration of RA, the corresponding ethanol–water percentage solution was used as a control to check the absence of antibacterial activity. The minimum inhibitory concentration (MIC), which is the lowest concentration at which there was a clear zone surrounding the wells, was then determined. Experiments were performed in triplicates and the average MIC was calculated.

### Cyclic voltammetry

Electrochemical measurements were performed by using the 797 VA Computrace potentiostat (Metrohm GmbH, Essen, Germany). A three-electrodes system was used through-out the study. A platinum electrode (working electrode, 3 mm in diameter), a platinum wire (counter electrode) and a Saturated Calomel Electrode were used. The working electrode was prepared by polishing on a micro polish cloth with water suspension containing alumina particles (50 nm diameter) then rinsed with water, ethanol, and acetone. Cyclic voltammetry experiments were conducted at room temperature in a glass cell containing 25 mL of acetonitrile with 0.1 M of tetrabutylammonium hexafluorophosphate Bu_4_NPF_6_ as supporting electrolyte.

### Spectrophotometric measurements

RA solution was prepared in the same conditions of the cyclic voltammetry to examine the absorption properties in the range 200–700 nm and to exclude possible interference. Measurements were made on a V-530 UV–Vis spectrophotometer (Jasco Corporation, Tokyo, Japan) with quartz cuvettes of 1.00 cm path length.

### Statistical analysis

Data from the colorimetric reduction of MTT was analyzed by cell type, RA concentration, and time of incubation after RA treatment. The data were normalized against the values obtained with the blank (0 µM RA). A two-way ANOVA was performed using data from the 72-h incubation time point with both cell types and RA concentration as group identifiers. Post-hoc Tuckey’s HSD test was used to determine significant differences seen between individual groups.

### Statement of compliance

Cultivated plants were collected in accordance with Lebanese American University and Ministry of Agriculture. Experimental research on plants and collection of plant materials were performed in accordance with relevant institutional, national, and international guidelines and legislation.

## Results and discussion

**Figure 1:**
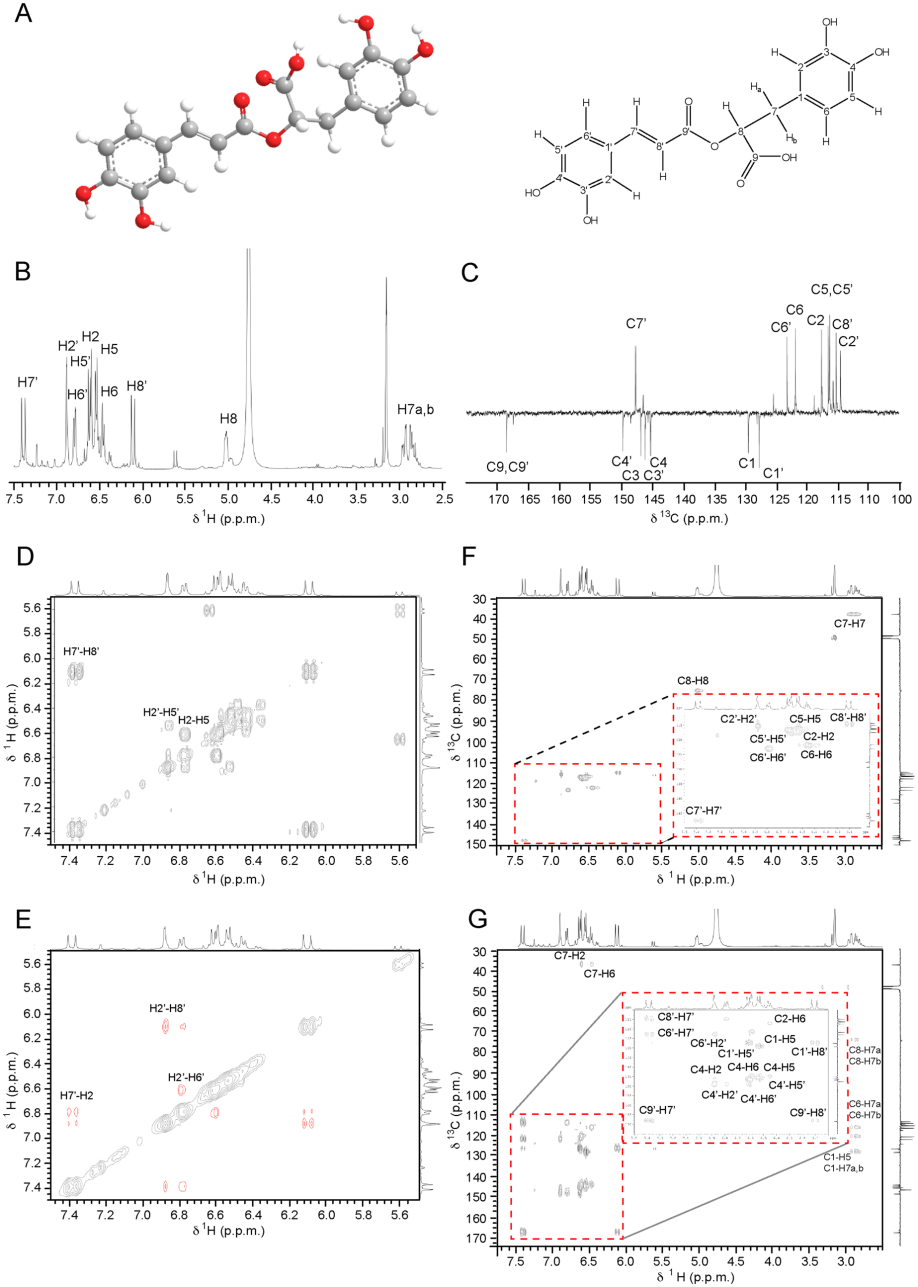
Structure elucidation of rosmarinic acid (RA) by 2D NMR spectroscopy. (**A**) Structure of RA. (**B**) 1D ^1^H NMR and (**C**) 1D ^13^C NMR spectra of RA. (**D**) 2D ^1^H-^1^H COSY and (**E**) 2D ^1^H-^1^H NOESY spectra of RA. (**F**) 2D ^1^H-^13^C HSQC and (**G**) 2D ^1^H-^13^C HMBC spectra of RA.

After Soxhlet extraction from Lemon Balm leaves, RA was first recovered from the extracts by solubilizing the crystals with methyl tert-butyl ether (MTBE), an optimized column chromatography protocol (Sephadex LH 20, methanol mobile phase) was then used for purification; and structure elucidation was accomplished by 1 dimensional (1D) and 2 dimensional (2D) NMR spectroscopy. The structure of RA (C_18_H_16_O_8_, Fig. 1) features two unsaturated 6-membered rings (C1–C6 and C1ʹ–C6ʹ), a double bond (C7ʹ–C8ʹ), four hydroxyl groups (C3, C4, C3ʹ, C4ʹ), an ester group (C9ʹ), and a carboxyl group (C9). ^1^H NMR spectrum of RA dissolved in CD_3_OD shows residual peaks at δ 4.75 ppm and δ 3.15 ppm corresponding to water peak and to methanolic CH_3_, respectively (Fig. 1). 9 CH groups, 1 CH_2_ and 8 quaternary carbons C_q_ were all visible on the ^13^C spectrum of RA (Fig. 1). The residual peak appearing at 48.99 ppm is the deuterated methanol. The 2D ^1^H–^1^H COSY correlation spectrum of RA displays information about J-coupled pairs of protons and usually designates that the protons are on adjacent carbons, 2 or 3 bonds away. To access information about pairs of protons apart through space up to 5A, we measured the 2D ^1^H–^1^H NOESY correlation spectrum of RA. We also measured 2D ^1^H–^13^C HSQC and 2D ^1^H–^13^C HMBC spectra to access direct proton–carbon J-couplings. The observed proton-proton coupling (*J* = 15.9 Hz) in the ^1^H NMR spectrum of RA shows two doublets (7.39 and 6.11) referring to a pair of *trans-*olefinic protons. Also, the aromatic region contains two ABX-spin systems assigned to two sets of protons of the 3,4-dihydroxyphenyl unit. All chemical shifts are in line with previously published data^42,43^ and are reported in Table 1.

**Table 1:** ^1^H NMR and ^13^C NMR chemical shifts of RA.

Position	^1^H	^13^C	Position	^1^H	^13^C
1	–	129.4	1ʹ	–	127.6
2	6.59	117.5	2ʹ	6.88	114.5
3	–	146.1	3ʹ	–	146.8
4	–	145.2	4ʹ	–	149.7
5	6.54	116.5	5ʹ	6.61	116.2
6	6.47	121.8	6ʹ	6.79	123.1
7a,b	2.85, 2.93	37.9	7ʹ	7.39	147.6
8	5.01	74.8	8ʹ	6.11	115.2
9	–	167.4	9ʹ	–	168.5

**Figure 2:**
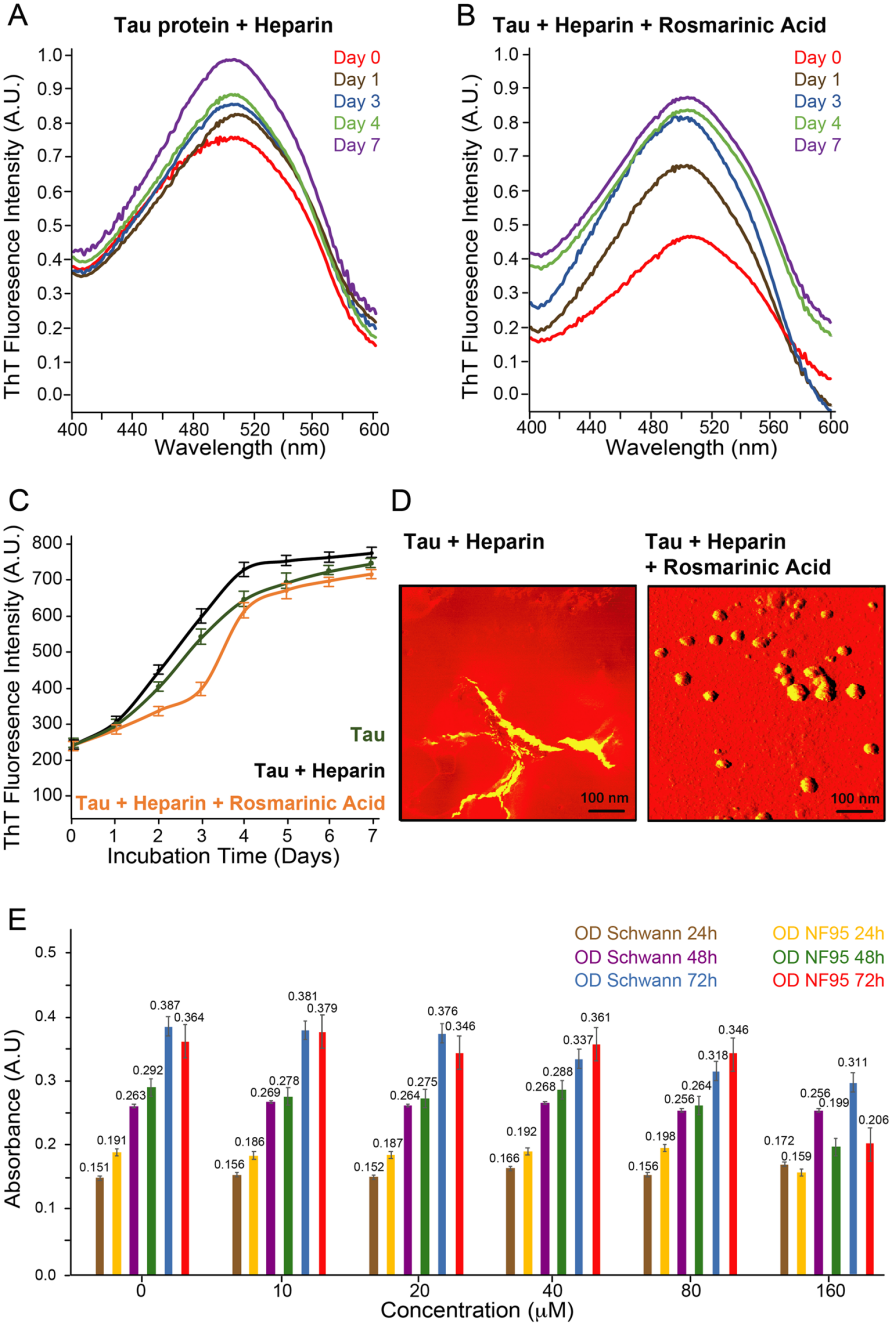
Anti-aggregation and anti-cancer effects of RA. Thioflavin T (ThT) fluorescence spectra of 50 μM Tau protein were measured after incubation with (**A**) 12.5 μM heparin alone or in presence of (**B**) 100 μM RA at 37 °C. Fluorescence was recorded at λ_excitation_ = 412 nm and λ_emission_ = 490 nm over a period of 7 days. (**C**) The ThT assay compares PHF assembly of free Tau (green) and in presence of heparin alone (black) or with RA (orange). It is evident that RA delays PHF assembly. Error bars were estimated from samples measured in triplicates. (**D**) After 7 days incubation, AFM images of Tau protein with heparin without and with RA were acquired (scale bar 100 nm). (**E**) The influence of RA in cellular viability was measured through mitochondrial reductase activity by MTT assay. NF95 and Schwann cells were treated with different concentrations of RA (10 to 160 μM) and incubated for different times prior to MTT treatment. Significant viability reduction was observed in NF95 cells (brown) exposed to 160 μM of RA for 72 h (p value: < 0.01) in comparison to Schwann cells (grey).

We then screened for the inhibition of PHF aggregation using full length Tau protein (Fig. 2). This construct aggregates reliably at low micromolar concentrations in presence of the polyanionic cofactor heparin and resembles Alzheimer’s PHFs in terms of fiber morphology and beta-sheet content^44,45^. The results described here were obtained by measuring the extent of aggregation via thioflavin T (ThT) fluorescence upon its binding to PHFs^46^. In this assay, we performed polymerization of full-length Tau at 37 °C in presence and absence of RA over a period of 7 days. RA was able to reduce the PHF-specific signal of ThT fluorescence by 60% and delay PHF assembly during the incubation period (Fig. 2). Previous studies on the assembly of PHFs revealed higher order Tau structures from stepwise association of core oligomeric units of ~ 15–25 nm in size and comprised of an assembly of 15 to 40 Tau monomers^47–49^. After 7 days incubation, we inspected the heparin-mediated PHF assembly in absence and presence of RA by contact mode Atomic Force Microscopy AFM (Fig. 2). Consistent with previous findings^48^, fibers with diverse morphologies were visible thus confirming the formation of Tau fibrils from soluble monomers upon incubation with heparin. On the other hand, no fibrils were visible in presence of RA but instead, spherical oligomeric assemblies were dominant, similar to what we previously identified^15^. Taken together, these findings suggest that RA delays PHF formation and effectively disassembles fibril assembly by redirecting them to oligomers.

Next, we probed the anticancer effects of RA in Neurofibromatosis. The influence of RA in cell viability and proliferation was measured by the mitochondria reductase enzyme activity through the MTT assay to test its inhibitory effects in plexiform neurofibroma tumor cells NF95. The MTT assay was performed in both the NF95 tumor cells and the non-tumor Schwann cells (glial cells of the peripheral nervous system as non-tumor control cells). Different concentrations of RA ranging between 10 and 160 μM did not result in any significant difference in Schwann cells (p-value: 0.7) or between the two different cell lines (p value: 0.25) but located significant differences within the NF95 cells (p value: 0.002). The Tuckey’s HSD post hoc analysis within the NF95 cells found that 160 µM RA incubated at 72 h prior to MTT addition had a significant difference (p value: < 0.01) with all other RA concentrations used (Fig. 2). These results indicate that RA effectively decreased mitochondrial activity in NF95 cells at 160 μM by causing a reduction in mitochondrial reductase activity in tumor cells after 72 h. The MTT assay is an indicator of cell viability and proliferation, both of which were reduced in NF95 cells.

**Figure 3:**
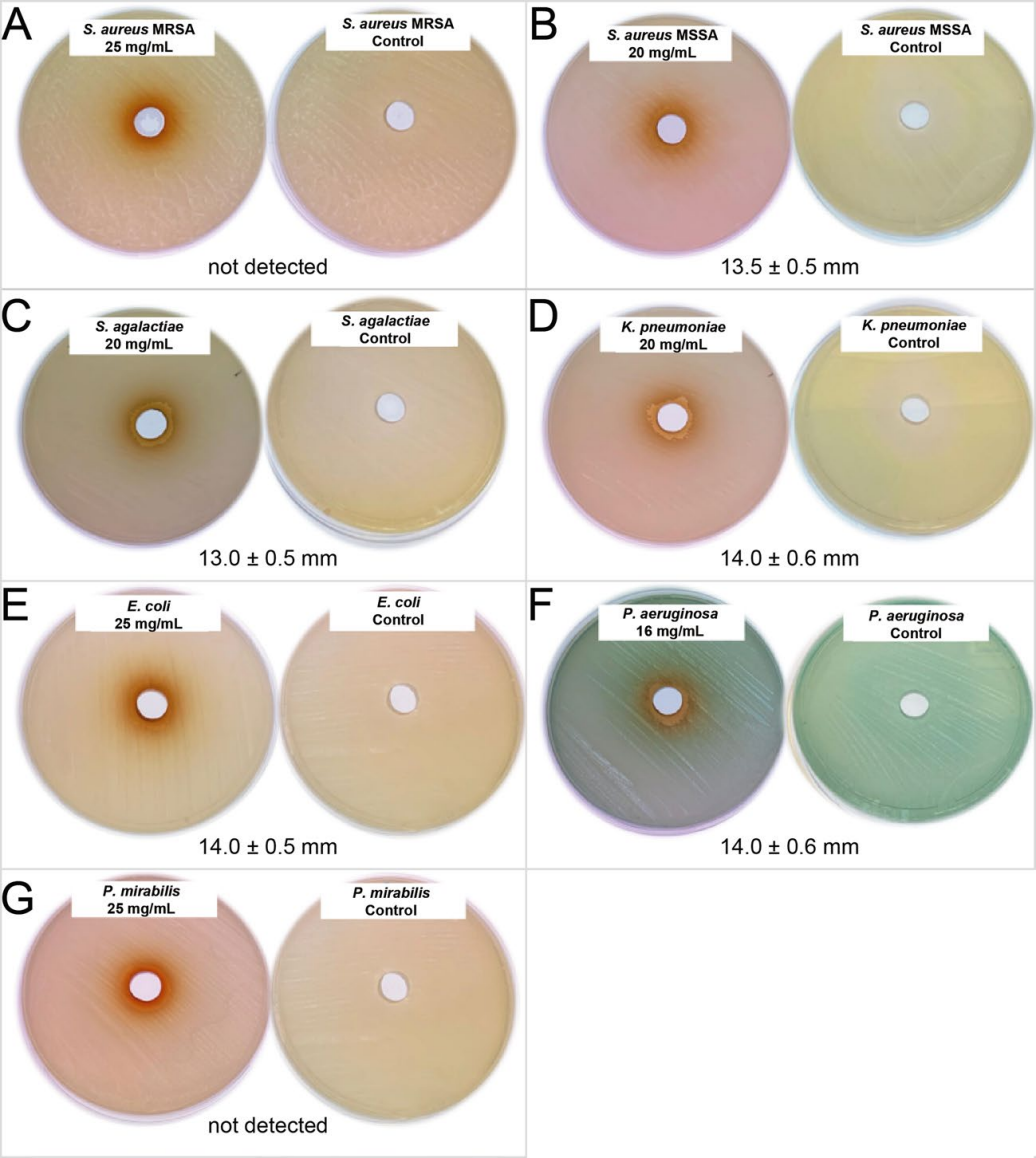
Anti-microbial activity of RA. Minimum Inhibitory Concentrations (MIC) values and diameter of inhibition of RA against three Gram-positive bacteria (**A**) *Staphylococcus aureus* MRSA, (**B**) *Staphylococcus aureus* MSSA (**C**) *Streptococcus agalactiae*, and four Gram-negative bacteria (**D**) *Klebsiella pneumoniae*, (**E**) *Escherichia coli*, (**F**) *Pseudomonas aeruginosa*, and (**G**) *Proteus mirabilis*. The diameters of the zones of inhibition (in mm) that correspond to the MIC of RA for each bacteria strain are reported.

To access the antibacterial effects of RA, we determined the Minimum Inhibitory Concentration (MIC) where a clear zone surrounding the wells appears. Figure 3 shows the MIC values of RA and the zones of inhibition. MICs ranged between 16 mg/mL to the limit concentration of 25 mg/mL and the zones of inhibition were around 13 mm for Gram-positive isolates and 14 mm for Gram-negative isolates. No inhibitory zone was observed in the negative controls. Hence, RA was not able to inhibit the growth of neither *S. aureus* MRSA nor *P. mirabilis* at the maximum tested concentration (25 mg/mL). This is in line with the fact that Gram-negative bacteria are more resistant to plant extracts or phenolic compounds than the Gram-positive bacteria, since they have a thick liposaccharide coated cell wall which could be impermeable to polar phenolic compounds^50,51^. The human pathogen *P. aeruginosa* was the most sensitive strain against RA (lowest MIC value = 16 mg/mL). On the other hand, the lowest antibacterial activity of RA was detected against *E. coli* (highest MIC value = 25 mg/mL). *S. aureus* MSSA, *S. agalactiae* and *K. pneumoniae* had the same level of sensitivity to RA (MIC = 20 mg/mL). Finally, 20 mg/mL of RA was able to inhibit the growth of *S. aureus* MSSA, *S. agalactiae* and *K. pneumoniae* isolates. Many studies have demonstrated the antibacterial activity of RA against *S. aureus* MRSA (MIC ranged between 0.39 and 3.13 mg/mL)^52^. The low MIC values are attributed to the synergetic effect of phenolic compounds in plant extracts. For instance, Benedec et al. interpreted that the antibacterial effect of lemon balm ethanolic extracts against *E. coli* and *S. aureus*, evaluated by the disk-diffusion assay, is due to the presence of RA (zones of inhibition 6 mm and 11 mm respectively)^53^. Similarly, Moreno et al. showed that RA-rich methanol extracts of rosemary have a significant antimicrobial activity against bacteria like *E. coli*, *S. aureus* and *K. pneumoniae*^54^. Many studies have also highlighted the susceptibility of *P. aeruginosa* to RA^55,56^. However, literature data are difficult to compare for several reasons such as the difference in antibacterial activity testing methods, difference in solvents used in the extraction process, the origin and purity of RA, and the synergistic effect when tested in plant extracts. Additionally, the results of confocal imaging revealed that RA acted at a genetic level and damaged the nucleoid of *P. aeruginosa*^57,58^.

**Figure 4:**
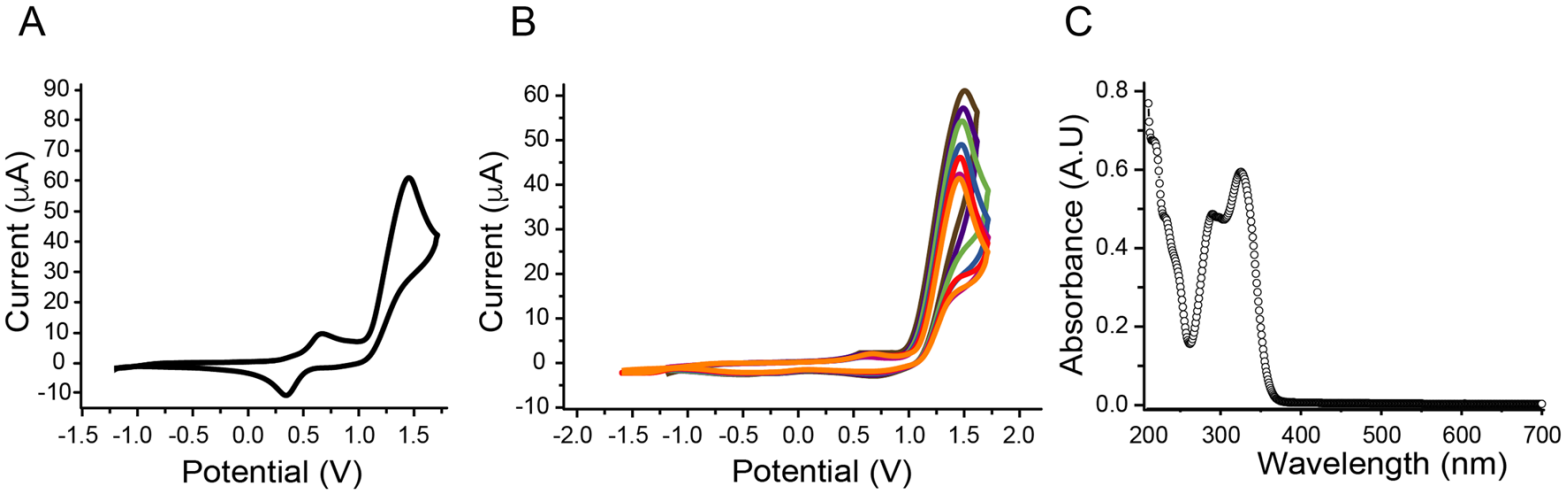
Electrochemical behavior of RA by cyclic voltammetry. (**A**) Cyclic voltammogram of RA in presence of 0.1 M Bu_4_NPF_6_ solution in CH_3_CN compared with internal standard ferrocene. (**B**) Cyclic voltammetry experiments recorded between − 1.5 and 1.6 V with sweep rate of 100 mV/s with a platinum working electrode. (**C**) UV–Visible spectrum of RA in CH_3_CN.

To shed lights on the mechanism of action of RA, we next probed its antioxidant activity through cyclic voltammetry, an electrochemical method of analysis for the assessment of redox-active correlation between the redox potential of the analyte and its capability to donate electrons^59^. The electrochemical properties of RA, which contains two oxidizable catechol moieties, were analyzed in acetonitrile for oxidation and reduction in the presence of tetrabutylammonium hexafluorophosphate Bu_4_NPF_6_ as supporting electrolyte (Fig. 4). The single trace CV of the compound shows an irreversible one-step two-electron oxidation wave with an anodic peak potential (E_pa_) of 0.785 V versus the redox system ferrocene/ferricenium ion as internal reference. Low oxidation potentials of a molecule imply that it has better electron donation and thereby can act as an antioxidant^60^. Interestingly, the electrochemical behavior of RA is like α-tocopherol, a very strong antioxidant used as food additive. The latter shows a single irreversible anodic wave with two electrons transferred per molecule^61^. The irreversibility of the anodic process in RA could be attributed to the slow proton exchange equilibrium in a polar aprotic solvent like acetonitrile^62^. Although such an irreversible wave is generally typical for highly reactive cation radical giving rise to electropolymerization^63^, however no electrodeposited material was observed under a constant applied potential of 1.6 V^64^. Also, application of recurrent potential scans up to 1.60 V to a solution of RA in CH_3_CN does not lead to the growing of any redox system that is typical for electrodeposition while no material was noticed to be attached onto the platinum anode surface. Anodic oxidation of RA has been widely studied, and conditions ranging from aqueous solutions of controlled pH media, to anhydrous organic solvents were applied to depict the electrochemical behavior of RA^65,66^. No reduction wave in the cathodic region could be detected within the electrochemical window of the solvent thus indicating a wide electrochemical band gap. This is further confirmed with the UV–Vis absorption spectrum of the compound in acetonitrile (Fig. 4). The solution spectrum shows an absorption maximum λ_max_ at 320 nm and main shoulder peaks at 290, 230, and 217 nm. The optical band gap estimated from the absorption edge value was calculated to be around 3.4 eV consistent with the colour of RA.

## Conclusions

We have isolated and purified RA from the dried leaves of Lemon balm. The structure elucidation of RA by NMR spectroscopy highlights the conformational and dynamic stability of the molecule. Interpretation of specific Tau fibrillization pathways may provide insights into disease mechanisms and reveal potential therapeutic targets for drug discovery. We also underlined the anti-aggregation potentials of RA against the formation of Tau filaments as deciphered by ThT assay and AFM. Using the MTT assay as an indicator of cell viability and proliferation, we then demonstrated that RA caused a reduction of mitochondrial reductase activity in NF95 tumor cells after 72 h. We furthermore investigated the antimicrobial activity of RA against Gram-positive and Gram-negative bacteria which are human pathogenic strains. In this intrinsic study, we have reached successful attempts to grasp highest degrees of purification, to effectively discard by-products, and to reliably elucidate the chemical structure of one of the most important antioxidants, anti-aggregation anti-inflammatory, and anti-microbial compounds. It is remarkable that RA has a very low toxicity and is quickly eliminated from the blood^67^ and therefore this natural product has broad practical applications in pharmacological investigations and in the treatment of numerous infections.

## Data Availability

The datasets used and/or analysed during the current study available from the corresponding author on reasonable request.
